# Prehospital time and mortality in polytrauma patients: a retrospective analysis

**DOI:** 10.1186/s12873-021-00476-6

**Published:** 2021-07-06

**Authors:** E. Berkeveld, Z. Popal, P. Schober, W. P. Zuidema, F. W. Bloemers, G. F. Giannakopoulos

**Affiliations:** 1 Department of Trauma Surgery, Amsterdam University Medical Center, location VUmc, De Boelelaan 1117, 1081 HV Amsterdam, The Netherlands; 2 Department of Anesthesiology, Amsterdam University Medical Center, location VUmc, De Boelelaan 1117, 1081 HV Amsterdam, The Netherlands; 3 Department of Trauma Surgery, Amsterdam University Medical Center, Location AMC, Meibergdreef 9, 1105 AZ Amsterdam, The Netherlands

**Keywords:** Prehospital time, Polytrauma patients, Mortality

## Abstract

**Background:**

The time from injury to treatment is considered as one of the major determinants for patient outcome after trauma. Previous studies already attempted to investigate the correlation between prehospital time and trauma patient outcome. However, the outcome for severely injured patients is not clear yet, as little data is available from prehospital systems with both Emergency Medical Services (EMS) and physician staffed Helicopter Emergency Medical Services (HEMS). Therefore, the aim was to investigate the association between prehospital time and mortality in polytrauma patients in a Dutch level I trauma center.

**Methods:**

A retrospective study was performed using data derived from the Dutch trauma registry of the National Network for Acute Care from Amsterdam UMC location VUmc over a 2-year period. Severely injured polytrauma patients (Injury Severity Score (ISS) ≥ 16), who were treated on-scene by EMS or both EMS and HEMS and transported to our level I trauma center, were included. Patient characteristics, prehospital time, comorbidity, mechanism of injury, type of injury, HEMS assistance, prehospital Glasgow Coma Score and ISS were analyzed using logistic regression analysis. The outcome measure was in-hospital mortality.

**Results:**

In total, 342 polytrauma patients were included in the analysis. The total mortality rate was 25.7% (n = 88). Similar mean prehospital times were found between the surviving and non-surviving patient groups, 45.3 min (SD 14.4) and 44.9 min (SD 13.2) respectively (*p* = 0.819). The confounder-adjusted analysis revealed no significant association between prehospital time and mortality (*p* = 0.156).

**Conclusion:**

This analysis found no association between prehospital time and mortality in polytrauma patients. Future research is recommended to explore factors of influence on prehospital time and mortality.

## Background

Traumatic injury accounts for 9% of all global fatalities [1]. Depending on injury severity, a prompt medical assessment, lifesaving onsite treatment and transportation to an appropriate trauma center are considered imperative to optimize survival rates [2, 3]. Therefore, constant improvement of Emergency Medical Services (EMS) care in resuscitation and rapid transportation might be of substantial impact on survival rates.

In the Netherlands, additional to the care provided by EMS, assistance from physician staffed Helicopter Emergency Medical Services (HEMS) can be requested to provide advanced specialized care and interventions on-scene, such as tracheal intubation, administration of advanced analgesia, chest tube placement and surgical procedures. Ultimately, the aim is to stabilize severely injured patients and rapidly transport them to an appropriate trauma center.

Despite these available methods, the optimal duration of prehospital time for severely injured patients is difficult to determine, as there is an assumption that a broad variety of factors could influence the prehospital time and mortality risk [4]. A previous systematic analysis has shown that for patients suffering penetrating or traumatic brain injury, a brief prehospital time would decrease mortality rates. This is in contrast with undifferentiated hemodynamically stable patients, who showed no increase in mortality odds with increasing prehospital time [5]. However, little empirical data exists considering severely injured patients in a prehospital setting characterized by both EMS or EMS and HEMS.

Therefore, this retrospective single center analysis aimed to examine the association between prehospital time and mortality in polytrauma patients in a Dutch level I trauma system. We hypothesize that short prehospital times reduce mortality rates and improve polytrauma patients’ outcome.

## Methods

### Study setting

This study was conducted in a Dutch trauma system characterized by prehospital care provided by EMS or both EMS and HEMS. Based on information from the initial call to the EMS dispatch center, often by a layperson, or upon request by EMS on-scene, HEMS can be dispatched. A HEMS crew consisting of a HEMS physician (trauma surgeon or anesthesiologist), HEMS nurse (Emergency Department’s or EMS nurse), HEMS pilot, and chauffeur can assist the EMS crew providing advanced lifesaving care on-scene. In most cases, after HEMS assistance on-scene, a patient is transported by ambulance with the HEMS physician on board, as distances to the nearest trauma center are often short and well accessible by road. In this study, the catchment area comprises of the northwest part of the Netherlands, covered by HEMS Lifeliner 1, including seven EMS dispatch centers over an area populated by 3.5 million inhabitants.

### Study design and data extraction

A retrospective analysis was performed based on data derived from the Dutch trauma registry of the National Network for Acute Care. Adult polytrauma patients (ISS ≥ 16) presented at the Amsterdam UMC, location VUmc (admitting approximately 1200 trauma patients annually of whom roughly 20% are considered polytrauma patients), over a 2-year period, were included.

Inclusion criteria consisted of treatment on-scene by EMS or both EMS and HEMS, followed by a direct transport to the trauma center. Patients below the age of 18 years, patients with missing data on the method of transportation to our center, patients with missing prehospital times and patients secondarily referred from surrounding hospitals were excluded from the analysis. Total prehospital time was calculated from when the EMS dispatch center received the initial call about the incident until the patient arrived at the trauma center. Patient characteristics, comorbidity (based on the American Society of Anesthesiologists Physical Status (ASA-PS)), mechanism of injury (MOI), type of injury (blunt or penetrating injury in which penetrating injury is composed of stabbing and shooting injury), HEMS assistance on-scene, prehospital Glasgow Coma Scale (GCS) and the injury severity score (ISS) were collected due to their potential confounding role on an association between prehospital time and mortality. The outcome measure was in-hospital mortality.

### Data analysis

Continuous variables were expressed as mean (standard deviation (SD)) or as median (interquartile range (IQR)) and were compared using independent sample t-tests or Mann Whitney U tests. Categorical variables were described as frequencies and percentages and compared using Pearson’s chi-squared tests. A binary logistic regression analysis was performed to investigate the association between prehospital time and mortality [6]. Initially, we used simple logistic regression to explore the unadjusted relationship. Next, to control for potential confounding, the following covariates were simultaneously included in the regression analysis: gender, age, comorbidity (based on the ASA-PS score), mechanism of injury, type of injury (blunt versus penetrating), HEMS assistance, prehospital GCS and ISS. These variables were selected based on previous literature, theoretical considerations and clinical relevance, rather than statistical significance in univariate testing, as currently recommended [7, 8]. To relax the assumption of a linear relationship between non-categorical independent variables and the logit of mortality, such variables were modelled as restricted cubic splines. Calibration and discrimination of the multivariable model were assessed using a Hosmer–Lemeshow test and the area under the receiver operating characteristic curve (AUROC), respectively [9].

Several explorative and sensitivity analyses were performed. First, to determine whether the association between prehospital time and mortality depends on other factors, specifically, ISS, prehospital GCS, comorbidity, injury mechanism, type of injury, as well as HEMS assistance, interactions between these factors and prehospital time were modelled. Second, instead of using spline variables, we modelled time as (A) a continuous variable and (B) as a categorical variable with cutoffs chosen at 35, 43 and 54 min to obtain 4 roughly equally sized groups. Third, the main analysis was repeated with multiple imputation of missing prehospital GCS scores. No power analysis was performed. The sample size was predetermined by the number of patients included in the Dutch trauma registry of the National Network for Acute Care over a 2-year period.

A *P*-value of < 0.05 was considered significant. Data were analyzed using IBM® SPSS® Statistics version 24.0 (IBM, New York, NY, USA) and STATA® version 16 (StataCorp LLC, College Station, TX, USA).

## Results

In total, 467 polytrauma patients were presented at our center during the study period. After exclusion of 125 patients due to missing data, an age below 18 years, objection to participate or secondary referrals, 342 patients were eligible for inclusion in the analysis.

The total study population consisted predominantly of male patients (67.5%), with a mean age of 52.1 (SD 20.5) years (Table 1). The majority of the injuries were caused by blunt trauma (94.2%), mainly due to traffic accidents. A median ISS of 22.0 (IQR 17.0–26.3) was found, with a total mortality rate of 25.7% (n = 88).

**Table 1:** Patient and prehospital characteristics

Variable	Survivors (n = 254, 74.3%)	Non-survivors (n = 88, 25.7%)	Total (n = 342)	*p*-value
Prehospital time, mean (SD)	45.3 (14.5)	44.9 (13.2)	45.2 (14.1)	0.501
Gender, male (%)	68.9	63.6	67.5	0.364
Age, mean (SD)	49.0 (19.2)	61.1 (21.5)	52.1 (20.5)	< 0.001*
*Comorbidity (%)*				
ASA 1	65.7	42.0	59.6	< 0.001*
ASA 2	30.4	38.7	32.5	0.151
ASA 3,4	3.9	19.3	7.9	< 0.001*
*Mechanism of injury (%)*				
Traffic	46.9	38.6	44.7	0.182
Fall from height	36.6	40.9	37.8	0.474
Other	16.5	20.5	17.5	0.401
Type of injury, blunt (%)	94.5	93.2	94.2	0.653
HEMS assistance, Yes (%)	46.1	62.5	50.3	0.008*
Prehospital GCS, median (IQR)	13.5 (6.0–15.0)	3.0 (3.0–13.0)	11.0 (3.0–15.0)	< 0.001*
ISS, median (IQR)	20.0 (17.0–25.0)	25.0 (25.0–29.8)	22.0 (17.0–26.3)	< 0.001*

*ASA* American Society of Anesthesiologists Physical Status, *HEMS* physician staffed Helicopter Emergency Medical Services, *prehospital GCS* prehospital Glasgow Coma Scale, *ISS* Injury Severity Score; *SD* standard deviation

*Statistically significant (*p* < 0.05)

Overall, the mean prehospital time was 45.2 min (SD 14.1). Similar mean prehospital times were found between the surviving and non-surviving patient groups, 45.3 min (SD 14.4) and 44.9 min (SD 13.2) respectively (*p* = 0.819). However, significant differences were found for prehospital GCS (*p* < 0.001) and ISS (*p* < 0.001) between the surviving and non-surviving group.

### Mortality analysis

**Fig. 1:**
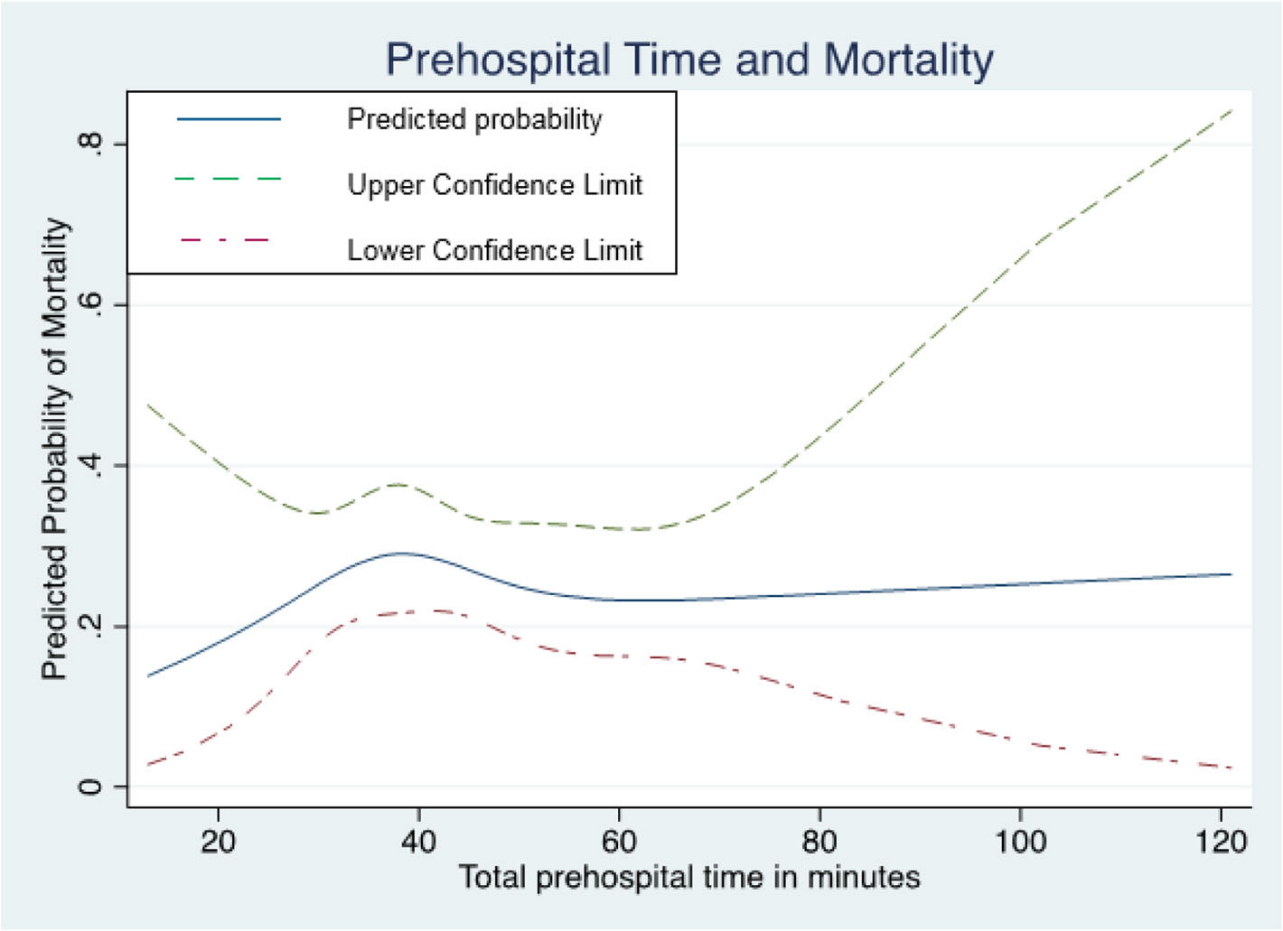
Association between prehospital time and mortality

**Fig. 2:**
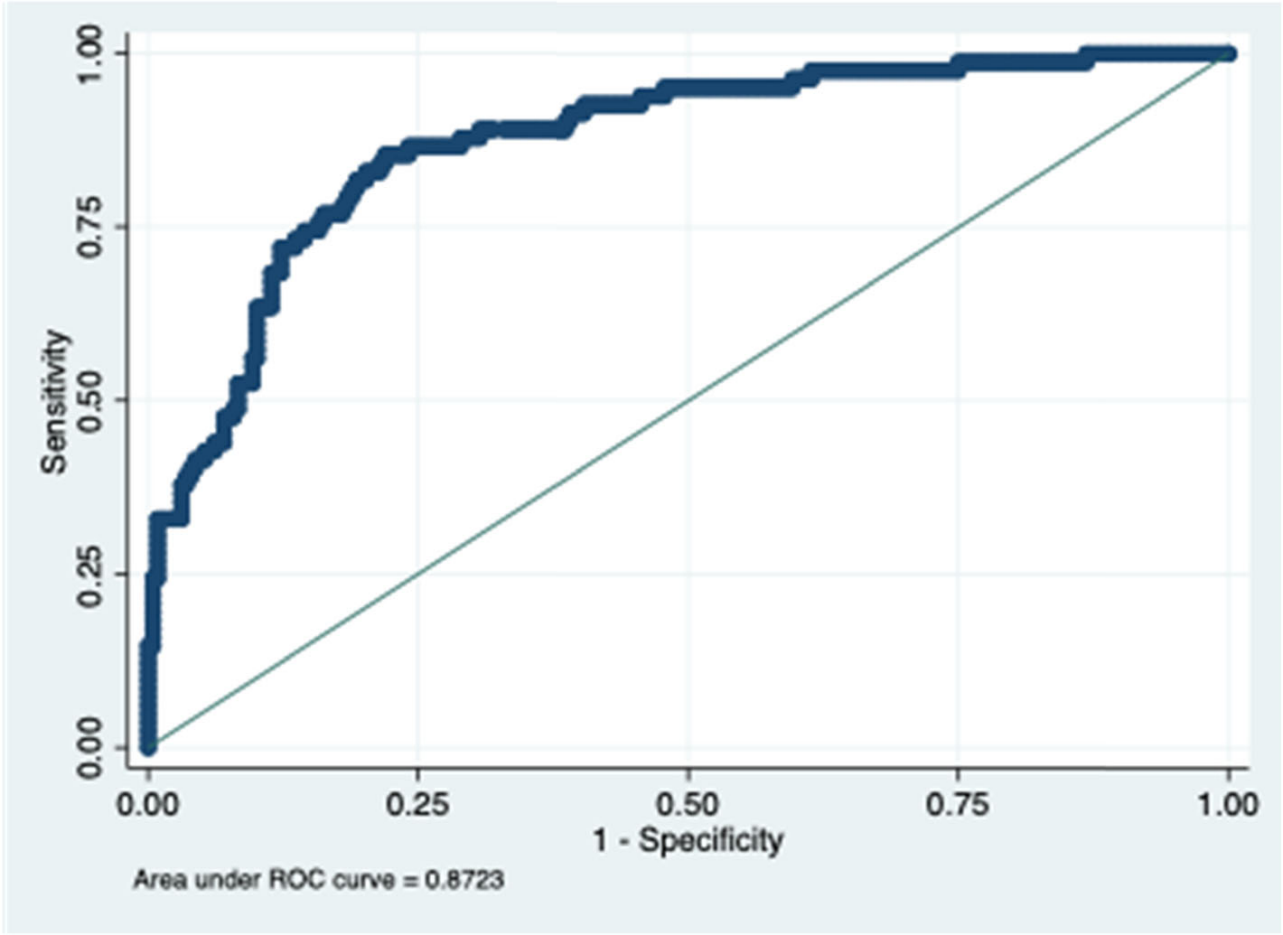
Receiver Operating Characteristic (ROC) Curve

The unadjusted association between prehospital time and mortality seemed to be non-linear in our sample (Fig. 1). However, the unadjusted logistic regression analysis actually did not provide evidence for any association between prehospital time and mortality (*p* = 0.754). Likewise, the confounder-adjusted analysis showed no association between prehospital time and mortality (*p* = 0.156). Significant relationships with mortality were observed for age, comorbidity, prehospital GCS and ISS (all *p* < 0.001). The model was characterized by an appropriate model fit (Hosmer–Lemeshow *p* = 0.551) and calibration as shown in Fig. 2 (AUROC = 0.872).

In the sensitivity analyses, the analysis after multiple imputation of the missing GCS values (n = 32) consistently also did not show any association between prehospital time and mortality. Also, when modeling time as continuous variable assuming a linear relationship or as a categorical variables, no association was found (*p* = 0.818 and *p* = 0.088, respectively). Moreover, no evidence for interactions between prehospital time and markers of injury characteristics, injury severity, comorbidities or HEMS involvement was observed.

## Discussion

This study analyzed 342 severely injured polytrauma patients, who had received advanced prehospital care and were transported directly to a level I trauma center. We hypothesized that a shorter prehospital time would decrease a patient’s mortality rate. However, in this analysis, no association between prehospital time and mortality was found.

Previous literature on the association between prehospital time and mortality in trauma patients is controversial. While some studies suggest that a short prehospital time seems beneficial in at least some patient categories [5, 10, 11], others—similarly to our results—did not observe an association between prehospital time and mortality [12–20]. Brown et al. found no association between prehospital time and in-hospital mortality in a polytrauma patient cohort in Australia, even not after stratification for a prehospital time equal to or above 60 min [21]. This is similar to Lerner et al., who, after adjustments for ISS > 15, patient demographics and treatment factors found no association between prehospital time and mortality [16]. In the prehospital phase, a well-used time frame is advocated by Klein et al. in German and Austrian trauma systems, emphasizing the performance of lifesaving treatment on-scene [22]. In addition, in a recent study conducted by Mills et al., with a cohort of patients who were transported with the highest EMS priority after a traffic accident, also no association between prehospital time and mortality was found [13].

In our study, the fact that we found no evidence for an association could be explained by several factors. First, distances to trauma centers are generally short in the Netherlands, and most patients in our region can be transported to our level I trauma center within fifteen to twenty minutes from any location. Indeed, we observed a mean prehospital time—including activation time, response time, on-scene time and transport time until arrival at the hospital—of only 45.2 min, which is relatively short compared to studies conducted in other trauma systems [23, 24]. It is thus possible that prehospital time is less relevant for the outcome within the rather short time-frame as observed in our study, but it could plausibly become increasingly important in systems in which patients have to be transported from remote areas.

Second, even though patients in this study population were all polytrauma patients as defined by an ISS ≥ 16, each individual patient, trauma mechanism and subsequent injury are unique, and thus, the group of polytrauma patients is highly heterogeneous. It is likely that a short prehospital time and quick in-hospital treatment is more important for some patients than for others, potentially explaining why overall no association was observed in the heterogeneous population. We have explored interactions between prehospital time and markers of injury characteristics or injury severity, but could not find evidence that the effect of time on mortality depends on any of these factors. Nonetheless, such interactions cannot be excluded, and it seems plausible based on previous literature that certain patients—such as those with penetrating injury or traumatic brain injury [5, 10, 11]—should be quickly transported to a trauma center. As it is often unclear which individual patient will versus will not benefit from quick transportation, we believe that it is generally prudent to avoid unnecessary delays and to initiate transport to a trauma center as soon as feasible.

Third, our trauma system is characterized by a wide array of treatment options for trauma patients. Stabilization is performed by highly trained and specialized EMS nurses, when required with the assistance of a physician-based HEMS crew. Potentially life-saving treatments, that otherwise would be reserved for the hospital setting, can be initiated on-scene. These treatments include advanced airway management, prehospital administration of blood products, inotropic or vasopressor support, antibiotic treatment for open fractures, or certain surgical procedures such as chest tubes and clamshell thoracotomies. Therefore, while HEMS involvement may prolong the prehospital time, it actually often shortens the time to advanced treatment [15–17]. This, in turn, may at least partially explain why a longer prehospital treatment time is not necessarily associated with worse outcomes in our patient cohort.

In this study, a limiting factor was the retrospective observational nature of the dataset with all the inherent limitations, such as confounding and missing data on prehospital GCS scores. However, the amount of missing values was moderate (< 10%), and sensitivity analysis with multiple imputation of prehospital GCS scores provided consistent results. In addition, we rigorously adjusted for potential confounders. Consistent with the literature, increased age, comorbidity, a low prehospital GCS and high ISS showed a significant association with mortality. Our analysis controlled for all of these factors, as well as for other potential confounders such as gender, type and mechanism of injury, and HEMS assistance. Nonetheless, residual confounding due to unobserved variables – such as hemodynamic, respiratory and physiologic parameters that are known to affect mortality in trauma patients [5, 10, 11, 25]—cannot be excluded. Furthermore, excluding patients with missing or incomplete prehospital time and reporting solely on in-hospital mortality, not taking into account prehospital mortality, might have caused a certain degree of selection bias. The single center design is another limitation. Our findings—likely at least partially explained by specific characteristics of our prehospital operation such as availability of highly trained EMS nurses and HEMS physicians in combination with short distances to trauma centers—do not readily generalize to settings with other logistic and geographic characteristics.

## Conclusion

This retrospective analysis based on polytrauma patients from a level I trauma center found no association between prehospital time and mortality. Nonetheless, our data do not exclude that individual patients may benefit from short prehospital times, and we suggest avoiding unnecessary delays in transporting patients to an appropriate trauma center. Future research is recommended to explore additional factors of influence on prehospital time and mortality, especially focusing on physiologic parameters in the severely injured patients.

## Data Availability

The datasets used and/or analyzed during the current study available from the corresponding author on reasonable request.
